# The lipid–inflammation axis in rosacea: mechanistic insights and therapeutic implications

**DOI:** 10.3389/fimmu.2026.1820173

**Published:** 2026-05-04

**Authors:** Xiaoyu Zhang, Tao Ning, Yanyan Feng

**Affiliations:** 1 Department of Dermatology, Sichuan University affiliated Chengdu Second People’s Hospital, Chengdu, China; 2 Chengdu Second People’s Hospital, Chengdu, China; 3 West China School of Medicine, Sichuan University, Chengdu, China

**Keywords:** rosacea, inflammation, lipid metabolism, lipids, pathogenesis

## Abstract

Rosacea is a chronic inflammatory skin disorder primarily characterized by persistent facial erythema, episodic flushing, papules and pustules, as well as phymatous changes. Despite extensive research, the pathogenesis of rosacea has not yet been fully elucidated. Current evidence suggests that rosacea develops on the basis of genetic susceptibility and results from the complex interplay of multiple endogenous and environmental factors, including immune dysregulation, impairment of skin barrier function, microbial imbalance, metabolic disturbances, and neurovascular dysfunction. Due to its chronic, refractory, and recurrent nature, rosacea imposes a substantial physical and psychological burden on affected individuals and markedly compromises quality of life. In recent years, accumulating studies have demonstrated that patients with rosacea frequently present with metabolic comorbidities related to lipid metabolism disorders, such as dyslipidemia, diabetes mellitus, and obesity. Notably, these metabolic abnormalities are reflected not only in circulating lipid profiles but also in alterations in the composition and proportion of facial sebum lipids. This review summarizes current evidence regarding abnormalities in serum lipid profiles and cutaneous lipid metabolism in patients with rosacea, and discusses the potential mechanisms by which dysregulated lipid metabolism may contribute to the initiation and progression of rosacea.

## 1 Introduction

Rosacea is a chronic inflammatory skin disorder that predominantly affects the central region of the face, involving the pilosebaceous units and the neurovascular system. Its main clinical manifestations include persistent facial erythema, episodic flushing, telangiectasia, and the formation of papules and pustules, often accompanied by subjective symptoms such as burning, stinging, and skin dryness. Based on clinical presentation, rosacea is classified into erythematotelangiectatic rosacea (ETR), papulopustular rosacea (PPR), phymatous rosacea, and ocular rosacea (1). Epidemiological studies indicate that the global prevalence of rosacea is approximately 5.1%, with a predilection for individuals aged 25–39 years (2). In the Chinese population, the prevalence is estimated to be around 3.48% (3). Although the pathophysiological mechanisms underlying rosacea remain incompletely understood, the disease is widely recognized as a multifactorial chronic inflammatory disorder. Its pathogenesis is thought to involve a complex interplay of genetic susceptibility, dysregulation of innate and adaptive immune responses, neurovascular dysfunction, imbalance of cutaneous and gut microbiota, and impairment of skin barrier function (4). In recent years, advances in metabolomics have drawn increasing attention to the role of metabolic disturbances in the development of rosacea. Metabolomic studies have revealed significant alterations in the abundance of multiple serum metabolites in patients with rosacea, suggesting the presence of systemic metabolic dysregulation (5). Moreover, accumulating evidence indicates that patients with rosacea have an increased risk of cardiovascular diseases and frequently present with metabolic comorbidities such as dyslipidemia and insulin resistance, further supporting a potential link between rosacea and systemic metabolic disorders.

## 2 Local lipid metabolic dysregulation

### 2.1 Alterations in skin barrier function and sebum lipid composition

Cutaneous lipids play a crucial role in maintaining skin barrier integrity and function. These lipids, including triglycerides, free fatty acids, wax esters, squalene, as well as smaller amounts of cholesterol and cholesterol esters, are primarily secreted by sebaceous glands and keratinocytes. Together, they form the skin surface lipid film, which contributes to barrier protection, immune defense, and the maintenance of epidermal homeostasis (6). Using thin-layer chromatography, researchers quantified facial skin surface lipids—such as cholesterol, free fatty acids, triglycerides, esters, and squalene—in patients with rosacea. The results showed that the relative total sebum content did not differ significantly between patients with rosacea and healthy controls (7). However, subsequent sebum lipidomic analyses using gas chromatography–mass spectrometry (GC–MS) in 25 patients with PPR revealed a distinct alteration in fatty acid composition. Specifically, the concentration of myristic acid (C14:0) was significantly increased in PPR patients, whereas levels of long-chain saturated fatty acids and monounsaturated fatty acids were decreased compared with controls. In addition, the composition of sebum fatty acids exhibited age- and sex-dependent variations (8). These findings suggest that, rather than changes in total sebum quantity, alterations in sebaceous fatty acid composition may play a more critical role in skin barrier dysfunction and the pathogenesis of PPR. An abnormal fatty acid profile may compromise the defensive capacity of the skin surface lipid film, thereby predisposing to or exacerbating cutaneous inflammation. Recent studies have further demonstrated that the composition and structural characteristics of skin surface lipids—including triglycerides (TAGs), diacylglycerols (DAGs), lysophosphatidylcholines (LPCs), and phosphatidylcholines (PCs)—differ significantly between patients with rosacea and healthy individuals, and are positively correlated with transepidermal water loss (TEWL) (9). Changes in facial skin lipids in rosacea are shown in **Table 1**. However, these studies did not specifically address differences in lipid composition between PPR and ETR subtypes. Notably, accumulating evidence indicates that the use of ceramide-containing moisturizers can effectively restore skin barrier function and is beneficial for patients with rosacea (10).

**Table 1:** Changes in facial skin lipids in rosacea.

Rosacea subtype	Types of lipids	Lipid Change Trend
PPR	Myristic acid (Cl4:0)	↑
	cis-11-Eicosenoic acid (C20:1)	↓
	Arachidic acid (C20:0)	↓
	Behenic acid (C22:0)	↓
	Tricosylic acid (C23:0)	↓
	Lignoceric acid (C24:0)	↓
ETR, PPR	LPC	↑
	PC	↑
	DAG	↑
	TAG	Different based on chain length
	Docosahexaenoic acid	↓

↑ indicates a statistically significant increase compared with the control group; ↓ indicates a statistically significant decrease compared with the control group.

### 2.2 Lipid metabolic dysregulation and imbalance of the cutaneous microbiome

Alterations in the facial skin microbiome of patients with rosacea are closely associated with immune mechanisms underlying disease pathogenesis. Rosacea is characterized by impaired skin barrier function, manifested by a significantly elevated skin surface pH and increased TEWL. These changes have been shown to induce alterations in both the abundance and composition of microbial communities colonizing the skin. Barrier dysfunction and microbiome dysbiosis act synergistically, thereby contributing to the initiation and progression of rosacea.Studies have demonstrated a markedly increased abundance of *Malassezia* species on the facial skin of patients with rosacea (11). *Malassezia* secretes lipases that hydrolyze sebum lipids, such as triglycerides, into free fatty acids. Saturated fatty acids are preferentially consumed by *Malassezia*, whereas irritant unsaturated fatty acids—predominantly oleic acid—accumulate on the skin surface, further accelerating skin barrier disruption and promoting inflammation (12). *Cutibacterium acnes* (formerly *Propionibacterium acnes*) metabolizes sebum into free fatty acids that help maintain a weakly acidic skin surface pH, thereby inhibiting colonization by opportunistic pathogens and exerting a protective role in healthy skin (13). However, follicular biopsy studies have shown that *C. acnes* does not play a primary pathogenic role in rosacea. Similar to observations in atopic dermatitis and psoriasis, the abundance of *C. acnes* on the facial skin of patients with rosacea is significantly reduced compared with healthy controls. This reduction is thought to weaken its protective effects on skin barrier function, thereby contributing to disease development (14).

In addition, *Demodex* mites represent an important component of the facial skin microbiome. Under physiological conditions, *Demodex* exists at low densities within human pilosebaceous units, where it feeds on sebum and contributes to immune tolerance. In patients with rosacea—particularly those with PPR—the density of *Demodex* mites is markedly increased. Metabolic products derived from *Demodex*, including proteins and lipids, may act as pathogen-associated molecular patterns (PAMPs), leading to activation of the TLR2–LL-37 signaling pathway. This activation may trigger inflammatory cascades, resulting in erythema and pathological angiogenesis (13).

### 2.3 Abnormal lipid synthesis and adiponectin

Emerging evidence indicates that key structural components responsible for maintaining skin barrier integrity are markedly altered in patients with PPR. These alterations include abnormalities in the formation of the cornified envelope and intercellular lipid lamellae, as well as structural changes in desmosomes and tight junctions. Proper formation of the intercellular lipid lamellae is essential for normal stratum corneum development. Among the critical regulators of lipid synthesis and transport, lipoxygenases (ALOX12B and ALOXE3) and the lipid transporter ATP-binding cassette subfamily A member 12 (ABCA12) play indispensable roles. Notably, the mRNA expression level of ABCA12 is significantly reduced in skin samples from patients with PPR compared with healthy controls, highlighting the presence of impaired intercellular lipid synthesis in PPR (15).

**Figure 1:**
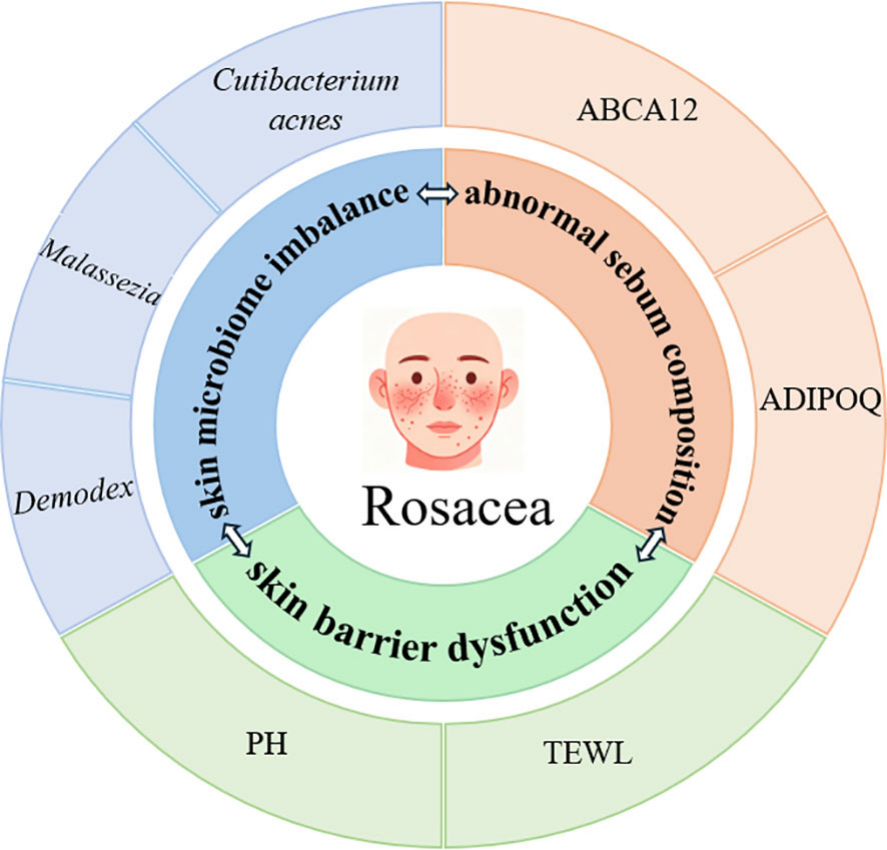
Cutaneous lipid metabolism in rosacea. The schematic representation of local lipid metabolism in rosacea indicates that the cutaneous microbiome—including *Cutibacterium acnes*, *Malassezia* species, and *Demodex* mites—together with impaired skin barrier function, may contribute to the initiation and progression of rosacea by utilizing facial sebum lipids as inflammatory mediators. It should be noted that, although the precise mechanisms have not yet been fully elucidated, dysbiosis of the facial microbiome, disruption of skin barrier integrity, and alterations in skin lipid composition are not independent processes. Rather, these factors are likely to be closely interconnected and may interact synergistically to promote disease development and progression.

In addition to structural lipid abnormalities, adipokine dysregulation has been implicated in the pathogenesis of rosacea. The expression of adiponectin (ADIPOQ) mRNA is significantly decreased in lesional skin of patients with rosacea (16). Adiponectin is one of the most extensively studied adipokines and plays a pivotal role in the regulation of glucose, lipid, and energy metabolism. It exerts anti-inflammatory effects in various inflammatory disorders, including psoriasis and atherosclerosis, partly through inhibition of ribosomal protein S6 phosphorylation. Joong et al. demonstrated that adiponectin expression is markedly lower in rosacea lesions than in non-lesional skin and healthy controls (16). Mechanistically, adiponectin suppresses phosphorylation of S6, a downstream marker of the mTORC1 signaling pathway, in the epidermis. In contrast, the antimicrobial peptide LL-37 induces keratinocytes to secrete pro-inflammatory mediators, such as nterleukin-6 (IL-6) and tumor necrosis factor-α (TNF-α), and concurrently inhibits adiponectin synthesis and secretion by subcutaneous adipocytes (17). These findings suggest that restoration of adiponectin signaling in the skin may represent a promising therapeutic strategy for rosacea. Local dysregulation of lipid metabolism in rosacea is shown in **Figure 1**.

## 3 Systemic lipid metabolic abnormalities

Although the association between rosacea and dyslipidemia has been widely documented, the mechanistic interactions between these conditions remain incompletely understood. Dyslipidemia is a well-established risk factor for cardiovascular and metabolic diseases and is generally indicative of a chronic low-grade inflammatory state. Notably, multiple studies have reported that patients with rosacea exhibit significantly elevated levels of systemic immune–inflammatory index (SII), erythrocyte sedimentation rate (ESR), and C-reactive protein (CRP) compared with healthy controls (18). These findings suggest that, beyond localized cutaneous inflammation, rosacea may be accompanied by a systemic inflammatory response, thereby providing a potential biological basis for its comorbidity with various systemic disorders. Metabolomic investigations have revealed pronounced disturbances in serum metabolism among patients with rosacea, characterized by elevated levels of glutamate and aspartate, as well as significant alterations in the abundance of fatty acids, organic acids, and carbohydrates (5). Importantly, amino acid metabolism is closely intertwined with lipid metabolism. Previous studies have demonstrated that serum glutamate concentrations are significantly associated with coronary artery disease and positively correlated with triglyceride levels (19). In addition, aspartate participates in lipid metabolism through the tricarboxylic acid (TCA) cycle (20). These aberrant amino acid metabolic signatures further support the presence of systemic lipid metabolic dysregulation in patients with rosacea.Recent serum proteomic profiling has further substantiated the involvement of metabolic alterations and neurofunctional dysregulation in the pathogenesis of rosacea. In these studies, downregulated differentially expressed proteins in rosacea patient serum were enriched in pathways related to lipid homeostasis and cholesterol metabolism. Moreover, proteins associated with flushing symptoms were predominantly involved in cholesterol metabolism and lipoprotein particle remodeling processes (21). These findings indicate that lipid metabolic disturbances may act synergistically with neurovascular regulatory abnormalities in rosacea.

Furthermore, Mendelian randomization analyses have provided genetic evidence supporting a potential causal role of lipid metabolism in rosacea susceptibility. Elevated plasma levels of sterol esters (27:1/22:6 and 27:1/15:0), phosphatidylethanolamines [O-18:2_20:4 and 18:0_20:4], and sphingomyelin (d34:0) were found to be significantly associated with a reduced risk of rosacea (22).

### 3.1 Low-density lipoprotein

The antimicrobial peptide LL-37 is markedly overexpressed in both lesional skin and serum of patients with rosacea. Excessive activation of this key effector of innate immunity may represent an important molecular mechanism underlying the association between rosacea and systemic comorbidities. LL-37 can activate classical inflammatory signaling pathways, including nuclear factor-κB (NF-κB), thereby not only exacerbating local cutaneous inflammation but also potentially promoting the development of a systemic pro-inflammatory state (23). Serum LL-37 levels have been shown to be significantly positively correlated with cardiovascular disease risk markers. In animal models, overexpression of humanized LL-37 increases susceptibility to cardiovascular disease. Mechanistically, LL-37 can bind to LDL, inducing conformational changes that enhance LDL uptake by macrophages and endothelial cells (24). This process may accelerate foam cell formation and atherogenesis, providing a plausible explanation for the increased risk of atherosclerotic cardiovascular disease observed in inflammatory skin disorders characterized by elevated LL-37 expression, including rosacea.Collectively, these findings suggest that LL-37 may serve as a mechanistic link between cutaneous inflammation and systemic lipid-related comorbidities, and highlight its potential utility as a novel biomarker for cardiovascular risk assessment in patients with inflammatory skin diseases.

### 3.2 High-density lipoprotein

Rosacea has a certain genetic predisposition, with specific human leukocyte antigen (HLA) alleles implicated in its pathogenesis. HLA-DQB1 is a class II gene within the HLA system, primarily involved in immune recognition. Studies have identified HLA-DQB1*03:03, HLA-DQB1*04:02, and the haplotype HLA-DQB1*03:03/05:02 as susceptibility alleles for rosacea. Neutrophils and HDL have been shown to exert mediating effects on the association between HLA-DQB1*03:03 and the risk or severity of rosacea (25). Based on these findings, it is hypothesized that HLA-DQB1*03:03 may increase the risk or severity of rosacea by promoting neutrophil recruitment and aggregation, as well as by suppressing HDL expression levels. Notably, serum HDL levels are negatively correlated with LL-37 gene expression (26), suggesting that HDL may act as a protective factor in the pathogenesis of rosacea.

### 3.3 Cholesterol

Previous studies have demonstrated an association between LL-37 expression and hypercholesterolemia. Elevated levels of LL-37 protein may mediate increased serum α-defensin concentrations, thereby promoting the accumulation of total cholesterol (TC) and low-density lipoprotein cholesterol (LDL-C) in circulation (27). In the context of rosacea, various triggering factors—such as microbial infection and ultraviolet radiation—can induce overexpression of LL-37. LL-37, in turn, promotes activation of the nucleotide-binding oligomerization domain-like receptor protein 3 (NLRP3) inflammasome through multiple mechanisms. Activation of the NLRP3 inflammasome may lead to the release of IL-1β and IL-18, as well as structural alterations in lipoproteins, thereby impairing cholesterol catabolism and transport and ultimately resulting in abnormal cholesterol accumulation (28).

In addition, ocular rosacea is frequently underrecognized in clinical practice. It is characterized by erythema of the eyelid margin, telangiectasia, and meibomian gland dysfunction (MGD) (29, 30). Studies have shown that patients with MGD exhibit significantly increased levels of free cholesterol and cholesterol esters in meibomian gland secretions compared with healthy controls, suggesting that abnormal lipid secretion may play a critical role in the pathogenesis of MGD. Moreover, patients with moderate to severe MGD also display systemic dyslipidemia, characterized by elevated total cholesterol and HDL levels. Consequently, some researchers have proposed that ocular manifestations may serve as early clinical indicators for screening rosacea-associated lipid abnormalities and for considering early intervention with statin therapy (31).

### 3.4 n-3/n-6 polyunsaturated fatty acids

Linoleic acid (LNA) is a major component of n-6 polyunsaturated fatty acids (PUFAs). Recent studies have demonstrated that levels of linoleic acid and its metabolites are significantly elevated in patients with rosacea. Mechanistically, LNA activates peroxisome proliferator-activated receptor-γ (PPAR-γ), leading to reduced production of reactive oxygen species (ROS), increased ATP generation, and restoration of mitochondrial membrane potential. Through these effects, LNA promotes the repair of damaged mitochondria in keratinocytes and thereby contributes to the suppression of rosacea development and progression. A prospective cohort study based on the UK Biobank, combined with linkage disequilibrium score regression (LDSC) analysis, further confirmed a significant negative association between circulating LNA levels and rosacea risk (32), highlighting the considerable therapeutic potential of LNA in rosacea management.

γ-Linolenic acid (GLA) is another endogenous n-6 PUFA synthesized in the liver from linoleic acid via Δ6-desaturase. In addition to its lipid-lowering effects, GLA exhibits anti-inflammatory and antineoplastic properties and plays a role in restoring skin barrier function (33). Compared with healthy controls, serum GLA levels are significantly reduced in patients with ETR. Blood lipid changes in rosacea are presented in **Table 2**. Moreover, GLA concentrations are negatively correlated with disease severity as well as with anxiety and depression scores in patients with ETR (34). Given that ETR is frequently accompanied by neuropsychiatric symptoms such as anxiety, depression, and migraine, lipid metabolic dysregulation may represent a shared pathophysiological basis underlying the comorbidity between rosacea and mood disorders. Importantly, oral supplementation with GLA has been shown to alleviate facial erythema, reduce transepidermal water loss, and improve stratum corneum hydration in patients with rosacea, further supporting the protective role of GLA in disease management (33). n-3 polyunsaturated fatty acids (n-3 PUFAs) are essential fatty acids required for the maintenance of human health. Evidence suggests that dietary supplementation with n-3 PUFAs ameliorates LL-37–induced rosacea-like skin inflammation by inhibiting the TLR2/MyD88/NF-κB signaling pathway. Pathway enrichment analyses have revealed that the biological processes and signaling pathways regulated by n-3 PUFAs—including chemokine signaling pathways—closely overlap with key mechanisms involved in rosacea pathogenesis, particularly in ETR (35).

**Table 2:** Blood lipid changes in rosacea.

Lipid	Lipid change trend
LDL, TG, LDL, Tch	↑
HDL	↓
LNA	↑
γ-GLA	↓

↑ indicates a statistically significant increase compared with the control group; ↓ indicates a statistically significant decrease compared with the control group.

### 3.5 Short-chain fatty acids

The inflammatory state of rosacea is also subject to remote regulation via the gut–skin axis. Intestinal dysbiosis contributes to the pathogenesis of both rosacea and gastrointestinal disorders by impairing innate and adaptive immune functions of the intestinal epithelium (36). Through modulation of microbial exposure, the gut microbiota produces short-chain fatty acids (SCFAs), such as butyrate, which enter the systemic circulation and directly participate in the regulation of systemic immune responses (37). SCFAs, including butyrate and propionate, are capable of modulating immune responses mediated by macrophages and regulatory T cells (Tregs). These metabolites exert anti-inflammatory effects by inhibiting activation of the TLR2 and NF-κB signaling pathways, thereby reducing the expression of pro-inflammatory cytokines such as TNF-α, IL-6, and IL-8 (38). As key mediators of the gut–skin axis, SCFAs play an important role in attenuating cutaneous inflammation and maintaining immune homeostasis.

### 3.6 Oxidized lipids

**Figure 2:**
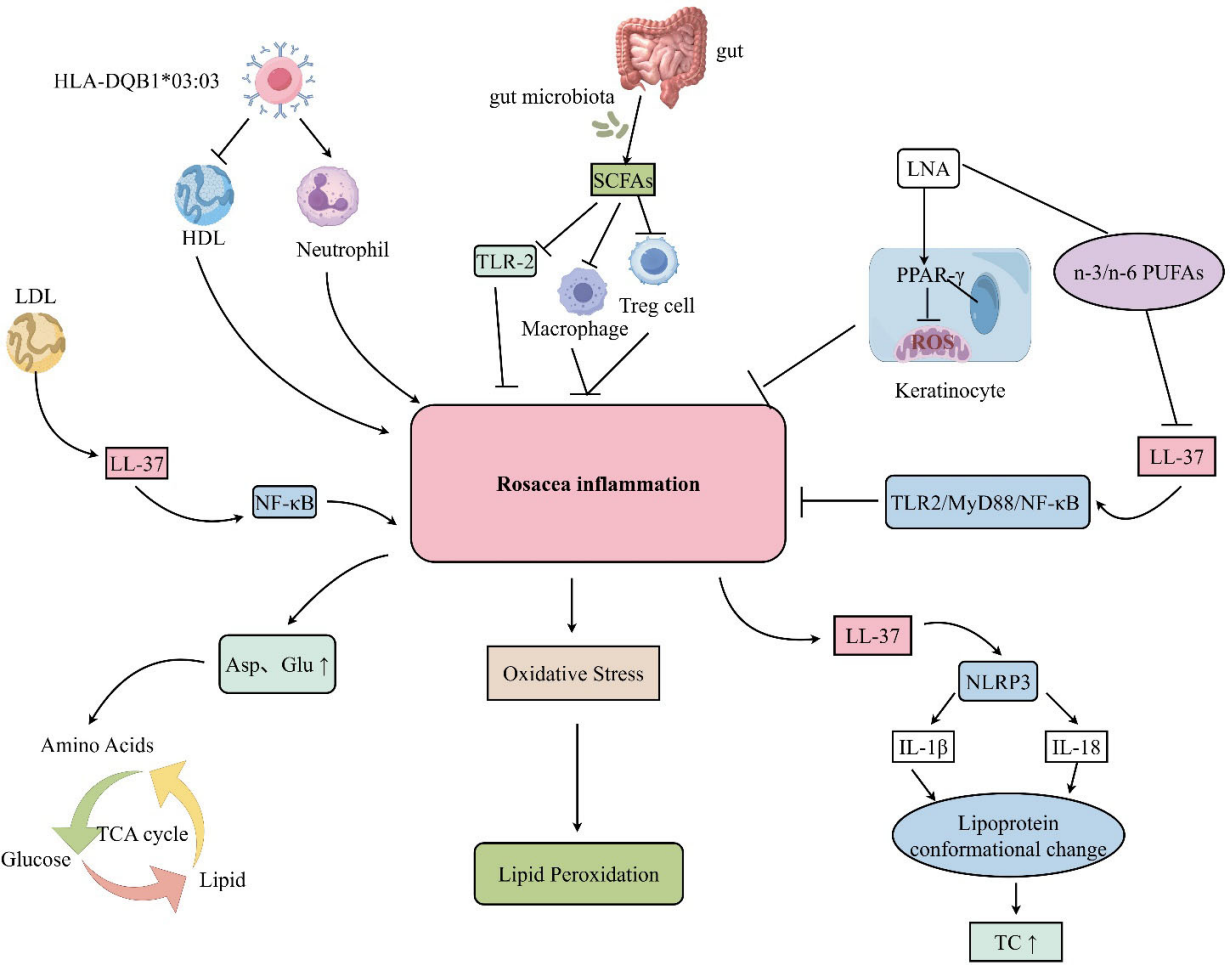
Systemic lipid metabolic abnormalities in rosacea. In rosacea, a complex lipid–inflammation axis exists at the systemic level, whose initiation and progression involve the synergistic effects of multiple factors, including genetic susceptibility (e.g., HLA-DQB1*03:03), gut microbiota dysbiosis, and oxidative stress. Concurrently, a variety of immune cells participate collectively, such as macrophages, T cells (especially regulatory T cells), neutrophils, and keratinocytes. At the molecular level, classic inflammatory signaling pathways including TLR2–LL-37–NF-κB and LL-37–NLRP3–IL-1β/IL-18 are activated, driving innate immune responses and inflammatory cascade amplification. LL-37 appears to function as a pivotal mediator in the lipid–inflammation axis of rosacea. It not only mediates the activation of immune-inflammatory pathways but also serves as a critical molecular bridge linking local inflammation in rosacea with systemic lipid metabolic abnormalities.

Accumulating evidence suggests that oxidative stress and lipid oxidation–driven inflammatory processes may contribute to the initiation and progression of rosacea. Compared with healthy controls, patients with rosacea exhibit markedly elevated levels of lipid hydroperoxides (39). These findings indicate an imbalance between oxidative and antioxidative systems in rosacea, resulting in enhanced oxidative stress and pronounced lipid peroxidative damage. Importantly, elevated serum lipid hydroperoxide levels may serve as a reliable indicator of increased oxidative stress in patients with rosacea, highlighting the potential involvement of oxidized lipids in disease pathophysiology. Systemic lipid metabolic dysregulation pathways in rosacea are depicted in **Figure 2**.

## 4 Therapeutic interventions and targets from a lipid metabolism perspective

Increasing evidence indicates that pharmacological treatments for rosacea are capable of reversing lipid metabolic abnormalities associated with the disease, further underscoring the pivotal role of dysregulated serum and/or facial sebum metabolism in rosacea pathogenesis.

### 4.1 Tetracyclines

Minocycline, a commonly prescribed agent for the treatment of rosacea, has been shown to reduce the concentration of oleic acid (C18:1n9) in sebum and to improve skin hydration. However, whether its therapeutic efficacy is directly mediated through modulation of the fatty acid profile remains to be fully elucidated (8). In addition, minocycline has been reported to alter the lipid composition of the meibomian glands in patients with rosacea-associated meibomian gland dysfunction, leading to reduced levels of free fatty acids and diacylglycerols (40). Doxycycline, another widely used tetracycline derivative, promotes sebocyte differentiation and enhances lipid production, potentially alleviating rosacea symptoms through regulation of sebaceous gland function and lipid metabolism (41). Collectively, these findings highlight the multifaceted effects of tetracyclines on lipid metabolism in rosacea, extending beyond their well-recognized anti-inflammatory and antimicrobial properties.

### 4.2 Retinoids

Low-dose oral isotretinoin has emerged as a potential therapeutic option for patients with moderate-to-severe refractory rosacea. Unlike its use in acne vulgaris, the therapeutic efficacy of isotretinoin in rosacea may be primarily attributed to alterations in sebum composition rather than a marked reduction in sebum production. Low-dose isotretinoin has been shown to modulate lipid composition by reducing the relative content of squalene in sebum, which may help normalize the excessively enhanced TLR2–mediated innate immune response observed in rosacea (42). In addition, isotretinoin not only alters the abundance of *Cutibacterium acnes* and *Malassezia* species within the cutaneous microbiome, but *in vitro* studies have also demonstrated that isotretinoin can reduce the density of *Demodex* mites in the skin (43). Through modulation of the skin microbiome, isotretinoin may further influence sebum composition and contribute to its therapeutic effects in rosacea.

### 4.3 Statins

Studies have demonstrated that topical statins may be effective in improving phymatous changes of rosacea, particularly nasal hypertrophy (44). In addition, patients with ocular rosacea, especially those with meibomian gland dysfunction and posterior blepharitis, may also benefit from statin therapy (31, 45). These effects are likely attributable to the pleiotropic properties of statins beyond their lipid-lowering action. Specifically, statins exert significant anti-inflammatory and immunomodulatory effects by reducing pro-inflammatory cytokines such as IL-6 and TNF-α and inhibiting macrophage-mediated inflammatory responses. They also possess antioxidant properties, mitigating oxidative stress through inhibition of oxidative enzyme activity and enhancement of antioxidant defenses. Furthermore, statins improve endothelial function and regulate vascular tone, and at higher doses, they can suppress vascular endothelial growth factor (VEGF) expression and angiogenesis. In addition, statins inhibit matrix metalloproteinases (MMPs), thereby attenuating inflammation and tissue remodeling (46). In view of the increased cardiovascular risk observed in patients with rosacea, statins may confer dual benefits by simultaneously improving cutaneous inflammation and systemic metabolic comorbidities. In particular, statin therapy may be especially beneficial for patients with ocular rosacea and those with concomitant dyslipidemia.

### 4.7 CKBA cream

The domestically developed CKBA (3-O-cyclohexanecarbonyl-11-keto-β-boswellic acid) cream has recently been approved for clinical investigation in rosacea, representing a promising therapeutic advance in this field. Mechanistically, CKBA directly inhibits acetyl-CoA carboxylase 1 and 2 (ACC1/ACC2), the rate-limiting enzymes governing *de novo* long-chain fatty acid synthesis, thereby targeting a central metabolic node in lipid biosynthesis. *In vitro* studies demonstrate that CKBA suppresses the differentiation of naïve CD4^+^ T cells into Th17 cells, markedly reduces IL-17A expression, and attenuates inflammatory cell infiltration (47). Through this coordinated modulation of lipid metabolism and adaptive immune responses, CKBA effectively dampens Th17-driven inflammation, providing a mechanistic basis for its potential to alleviate hallmark rosacea manifestations such as persistent erythema and flushing. Beyond its effects on fatty acid synthesis, CKBA has been shown to modulate multifunctional enzyme type 2 (MFE-2), a pivotal component of the peroxisomal β-oxidation pathway. By influencing this key metabolic regulator, CKBA exerts control over the lipid–inflammation axis, a critical interface linking metabolic reprogramming to inflammatory signaling. Notably, MFE-2 is essential for maintaining lipid homeostasis in microglia, and CKBA-mediated regulation of MFE-2 has been associated with suppression of pro-inflammatory cytokine production and restoration of metabolic equilibrium in neuroinflammatory contexts (48). These findings underscore the broader immunometabolic potential of CKBA and highlight lipid metabolic reprogramming as a rational therapeutic target in rosacea.

### 4.4 Dietary interventions

Given the presence of oxidative stress and lipid peroxidation abnormalities in patients with rosacea, supplementation with thiol-based antioxidants or enhancement of endogenous antioxidant capacity may help restore thiol/disulfide homeostasis, reduce lipid oxidative damage, and thereby attenuate inflammation (49). Both n-3 and n-6 polyunsaturated fatty acids (PUFAs) are essential fatty acids that must be obtained through dietary intake and are critical for normal growth and maintenance of health (50). A prospective cohort study involving 3,496 participants demonstrated that adherence to a Mediterranean dietary pattern rich in polyunsaturated fatty acids was associated with a reduced risk of incident rosacea (51). Diets high in dietary fiber and omega-3 fatty acids have also been shown to increase the production of short-chain fatty acids and the abundance of short-chain fatty acid–producing bacteria in the gut, thereby exerting systemic anti-inflammatory effects (52). Moreover, dietary supplementation with n-3 and n-6 PUFAs has been shown to exert anti-inflammatory and immunomodulatory effects in a range of dermatological conditions, including atopic dermatitis and photoaging (35). Combined supplementation strategies involving n-6 PUFAs (such as linoleic acid–rich oils and γ-linolenic acid as dietary supplements) together with n-3 PUFAs—particularly docosahexaenoic acid (DHA) and eicosapentaenoic acid (EPA)—may represent a promising adjunctive therapeutic approach for inflammatory skin diseases, including rosacea.

### 4.5 PPAR-γ

PPAR-γ plays a crucial negative regulatory role in immune and inflammatory responses. Previous studies have demonstrated that activation of PPAR-γ suppresses the secretion of pro-inflammatory cytokines, such as interferon-γ (IFN-γ) from macrophages and interleukin-12 (IL-12) from dendritic cells, thereby exerting significant anti-inflammatory effects in skin tissues (53). Owing to its broad immunomodulatory properties, PPAR-γ has been regarded as a potential therapeutic target in various inflammatory skin diseases, including psoriasis, atopic dermatitis, acne, and hidradenitis suppurativa. Notably, several polyunsaturated fatty acids, including linoleic acid, α-linolenic acid, as well as docosahexaenoic acid (DHA) and eicosapentaenoic acid (EPA), have been identified as natural ligands of PPAR-γ (54). Mechanistic studies have shown that linoleic acid can alleviate mitochondrial dysfunction by activating the PPAR-γ signaling pathway and suppressing excessive reactive oxygen species (ROS) production. Its potential role in regulating rosacea-associated inflammatory responses has been supported by preliminary experimental evidence (32). Clinically, thiazolidinediones—classical PPAR-γ agonists such as rosiglitazone and pioglitazone—are widely used in the treatment of type 2 diabetes mellitus. Whether these agents could exert therapeutic effects in rosacea by modulating immune-inflammatory responses and lipid metabolism remains to be elucidated through further basic and clinical investigations.

### 4.6 ACSL5

Transcriptomic analyses of rosacea lesions have identified acyl-CoA synthetase long-chain family member 5 (ACSL5) and very long-chain acyl-CoA dehydrogenase (ACADVL) as key target genes involved in lipid metabolism. Among these, ACSL5 is significantly upregulated in patients with ETR and co-localizes with markers of M1 macrophages. These findings suggest that ACSL5 may participate in M1 macrophage polarization through regulation of lipid metabolism, thereby triggering downstream inflammatory responses (55). Collectively, ACSL5 represents a potential therapeutic target for rosacea intervention.

## 5 Conclusion and outlook

In summary, both local sebaceous lipid alterations and systemic lipid metabolic dysregulation may play critical roles in the initiation and progression of rosacea. The emerging concept of a “lipid–inflammation axis” is likely to represent a central mechanistic framework underlying rosacea pathogenesis. Lipids not only influence the homeostasis of cutaneous and gut microbiota and modulate oxidative stress responses, but also directly participate in innate immune regulation and may interact with genetic susceptibility factors associated with rosacea. Accumulating evidence suggests that specific lipid species can either exacerbate or mitigate disease progression. However, the precise molecular mechanisms and regulatory targets involved remain incompletely understood. Future studies are warranted to elucidate the role of lipid metabolic reprogramming in rosacea pathophysiology and to identify novel therapeutic targets based on lipid-mediated signaling pathways.
